# Predicting Workability of a Low-Cost Powder Metallurgical Ti–5Al–2Fe–3Mo Alloy Using Constitutive Modeling and Processing Map

**DOI:** 10.3390/ma14040836

**Published:** 2021-02-09

**Authors:** Di Pan, Bin Liu, Rongjun Xu, Jingwen Qiu, Chunxuan Liu

**Affiliations:** 1 State Key Laboratory of Powder Metallurgy, Central South University, Changsha 410083, China; Pandycc2@csu.edu.cn (D.P.); xurongjun@csu.edu.cn (R.X.); 2 Hunan Provincial Key Laboratory of High Efficiency and Precision Machining of Difficult-to-Cut Material, Hunan University of Science and Technology, Xiangtan 411201, China; 3 Hunan Provincial Key Laboratory of Advanced Materials for New Energy Storage and Conversion, Hunan University of Science and Technology, Xiangtan 411201, China; 4 Hunan Gold-Sky Aluminum Industry High-Tech Co., Ltd., Luxi 416100, China; liuchunxuan315@126.com

**Keywords:** titanium alloy, low cost, workability, deformation behavior, constitutive modeling, processing map, deformation mechanism

## Abstract

A low-cost titanium alloy (Ti–5Al–2Fe–3Mo wt.%) was designed and fabricated by blended elemental powder metallurgy (BEPM) process. The high-temperature deformation behavior of the powder metallurgical Ti–5Al–2Fe–3Mo wt.% (PM-TiAlFeMo) alloy was investigated by hot compression tests at temperatures ranging from 700 to 1000 °C and strain rates ranging from 0.001 to 10 s^−1^. The flow curves were employed to develop the Arrhenius-type constitutive model in consideration of effects of deformation temperature, strain rate, and flow stress. The value of activation energy (Q) was determined as 413.25 kJ/mol. In order to describe the workability and predict the optimum hot processing parameters of the PM-TiAlFeMo alloy, the processing map has been established based on the true stress–true strain curves and power dissipation efficiency map. Moreover, microstructure observations match well with the analyses about deformation mechanisms, revealing that dynamic recovery and dynamic recrystallization are dominant softening mechanisms at relatively high temperatures. However, the kinking and breaking of microstructure prefer to occur at relatively low temperatures.

## 1. Introduction

Titanium alloys are important structural materials applied widely in aerospace, biomedical, and energy fields because of the characteristics of lightweight, excellent mechanical performance, good corrosion resistance, and biocompatibility [1,2]. However, the high cost of raw materials and complex manufacturing processes limit the further application of titanium alloys [3,4,5,6,7].

It is known that the powder metallurgy (PM) technique is relatively cost-effective because of its near-net-shape characteristic. Cost reduction in raw materials in titanium alloys mainly focuses on using cheaper substitution for those conventional expensive BCC phase stabilizers (i.e., V, Ta, and Nb). Some researchers consider Fe a promising substitution for V in α + β titanium alloys due to its relatively low cost and strong BCC phase stablishing ability [8,9]. It was reported that the addition of Fe could also help to enhance the sinterability of PM titanium components, thanks to its high diffusion rate in Ti [10,11,12]. On the other hand, the local segregation of Fe particles and the formation of a brittle TiFe phase could be prevented in the solid sintering process [13]. Recent literature [14] reported that higher yield strength (~1067 MPa) could be obtained by adding Fe in PM titanium alloy. Mo is also a cost-effective (compared with V) BCC phase stabilizer in titanium alloys [15]. Some references [16,17] indicated Mo could help obtain fine-grain microstructure during the sintering process and increase the strength, ductility, and creep resistance of titanium alloys. Furthermore, a relatively desirable combination of strength and ductility could be obtained in PM Ti–Mo binary alloys when the amount of Mo was 3 wt.% [11]. As a type of low-cost titanium alloys, TiAlFeMo series alloys have recently attracted more attention. Previous studies [18,19] of our research group have reported that 30% of the cost could be reduced in this series of alloys (compared with T–6Al–4V alloy). Furthermore, relatively high tensile strength (~1422 MPa) could be obtained in the hot rolled + annealed PM Ti–5Al–2Fe–3Mo alloy, and the elongation is measured as 8.5%, which showed a better coordination of strength and ductility [20].

However, previous works mainly focused on the improvement of mechanical properties of the TiAlFeMo series alloys. Systematic studies about the prediction of workability of this alloy have never been performed. Since the good plastic deformation ability of metal materials is the basis of their wide industrial applications, it is indispensable to reveal the deformation mechanism and optimize the thermomechanical parameter/process of this alloy during hot deformation.

In this work, a low-cost Ti–5Al–2Fe–3Mo (wt.%) alloy was fabricated using the blended elemental powder metallurgy (BEPM) technique. The flow behavior of the PM-TiAlFeMo alloy was investigated by hot compression tests using different thermomechanical processing parameters. A constitutive equation is a significant method that has been widely used to model the flow behaviors of metallic materials, helping to predict appropriate processing parameters using processing maps. Therefore, the Arrhenius-type constitutive model was established, and the processing map was also drawn to assess and predict the workability of the PM-TiAlFeMo alloy. The effects of the hot deformation parameters (testing temperature, deformation strain rate) on the flow behavior of the PM-TiAlFeMo alloy were discussed in detail, and microstructure observations were also performed before/after hot compression tests.

## 2. Materials and Methods

Raw alloy (Ti–5Al–2Fe–3Mo wt.%) was fabricated using pure-elemental powders (the details of these pure-elemental powders are listed in Table 1). The powders were blended in the correspondent wt.% and mixed in a double-cone powder mixer for 6 h (under argon as the protective gas). Then the uniformly blended powders were molded in a cold isostatic pressing system (180 MPa), followed by sintering at the temperature of 1300 °C for 2 h (in a vacuum of 10^−3^ Pa). The phase transformation temperature (T_β_) of this alloy is about 915 °C, calculated by the simulation software (Thermo-calc) [20] and determined by metallographic examination.

**Figure 1:**
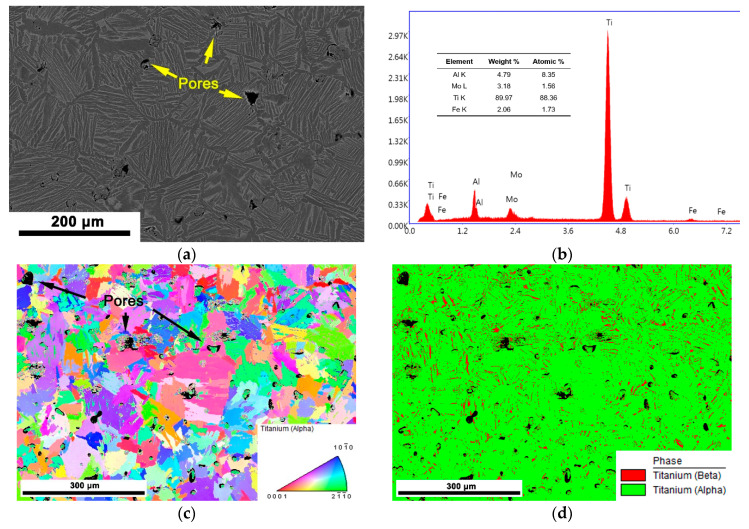
The microstructure of the as-sintered powder metallurgy (PM)-TiAlFeMo alloy: (**a**) backscatter scanning in E-beam (BSE) image; (**b**) EDS analysis; (**c**) IPF map; (**d**) phase image. (BSE and IPF are not generated from the same region.)

As shown in Figure 1, the microstructure of the as-sintered PM-TiAlFeMo alloy mainly consists of α phase (dark region in Figure 1a) and β phase (bright region in Figure 1a), and the α + β phase shows a nearly lamellar structure. The chemical composition of the as-sintered alloy measured by EDS (FEI, Hillsboro, OR, USA) (Figure 1b) was in agreement with the composition design. Some small pores can also be observed (marked by arrows in Figure 1a,c), the porosity of the as-sintered PM-TiAlFeMo alloy is about 9.8 % (measured by volume density and specific gravity). According to the EBSD result shown in Figure 1c, no distinct preferred orientation could be observed in the as-sintered alloy.

Cylindrical specimens of 8 mm × 12 mm (diameter × height) were cut from the as-sintered PM-TiAlFeMo alloy bar using a wire electrical discharge machine, then sanded by SiC abrasive sandpaper up to 2000 grit and polished with OPS–H_2_O_2_ diluent. Hot compression tests were performed on Gleeble-3500 thermal simulation testing system (DSI, New York, NY, USA). In order to homogenize the heat distribution, the specimens were heated at a rate of 3 °C/min and preserved for 3 min after target temperature. In addition, a thin graphite paper was placed between the specimen and holder to reduce the friction force. Water-quenching was performed for all the specimens to reserve the post-deformed microstructure after hot compression tests. The samples of microstructure observation were cut along the compressive axis (Marked as CA in figures). Microstructure observations of backscatter scanning in E-beam (BSE) and electron backscattered diffraction (EBSD) for the as-sintered and post-deformed PM-TiAlFeMo alloy were performed by FEI Helios NanoLab G3 UC Dual-Beam field emission scanning electron microscope (FEI, Hillsboro, OR, USA).

## 3. Results and Discussion

### 3.1. Flow Curves

#### 3.1.1. Flow Stress Characterization

**Figure 2:**
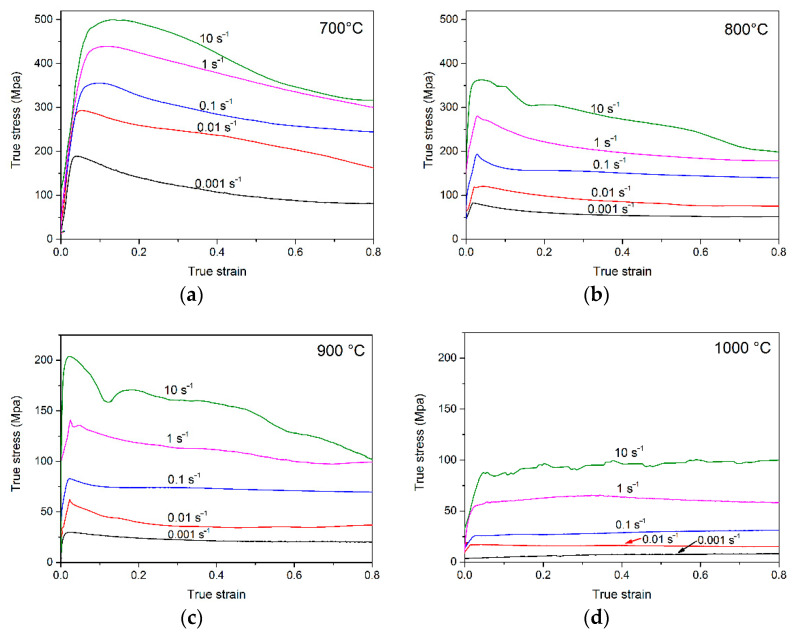
True stress–true strain curves of the PM-TiAlFeMo alloy deformed at different temperatures and strain rates: (**a**) 700 °C; (**b**) 800 °C; (**c**) 900 °C; and (**d**) 1000 °C.

The true stress–true strain curves at various deformation conditions are presented in Figure 2. Similar regularity could be observed at the temperatures below T_β_ (Figure 2a–c): the flow stress rises rapidly as the true strain increases before an obvious peak at the initial stage of hot compression (as the true strain ≤0.15). After the peak value, true stress exhibits a gradual falling with the increase in true strain, which is usually considered as a typical flow softening during compression. The rapid increase in flow stress may result from the formation of a huge number of dislocations at the beginning of the deformation, followed by the proliferation and tangles of dislocation [21]. The decrease in flow stress may be due to the dynamic recovery, dynamic recrystallization and dynamic globularization, which often occur as the true strain exceeds 0.15 and leads to the flow softening at the later stage of compression tests [22]. When the testing temperature increases beyond T_β_ (Figure 2d), no obvious peak value could be observed in the flow curves, the value of true stress tends to be steady. A similar result was obtained in the Ti600 alloy [23] and Ti-6242S alloy [24], which was considered as a typical steady-state flow. It was reported that the mechanisms of plastic deformation mainly depend on the crystal structure and the stacking fault energy [25]. In general, the steady-state flow is associated with dynamic recovery [23]. Hajari et al. [24] proposed that dynamic recovery always prefers to occur in materials of relatively high stacking fault energy. For titanium alloys, the β phase has higher stacking fault energy and diffusivity than the α phase, dynamic recovery is easier to occur when the testing temperature exceeds T_β_, which benefits the occurrence of steady-state flow at relatively high testing temperature. In addition, the deformation is mainly controlled by dislocation slipping for BCC Ti. When the compression tests were performed above the T_β_, the dislocations accumulated during deformation will be rearranged by the slipping and climbing of dislocations. Then, a dynamic balance could be reached between the work hardening and softening, where dynamic recovery and recrystallization often occur under this condition [26,27].

As shown in Figure 2, the peak values of true stress at a constant strain rate are negatively correlated with the testing temperatures. The average kinetic energy of atoms and strengthened function of thermal activation will increase when the deformation temperature rises, followed by the decrease in critical slip shear stress. Hence, the dislocation movement occurs more easily, leading to the decrease in peak value when the temperature rises. Meanwhile, the dislocation density will reduce, the structure softening of BCC Ti and grain coarsening will aggravate as the temperature increases, which also serves to weaken the work hardening during hot deformation.

Except for the testing temperature, the strain rate of hot compression is another factor of the flow behavior. The peak value increases as the strain rate increases from 0.001 s^−1^ to 10 s^−1^ at a given temperature. The main reason is the rapid work hardening resulting from the formation of a huge number of dislocations in the alloy when the strain rate increases. As shown in Figure 2b–d, a steady-state flow could be observed at relatively low strain rates, which means dynamic recovery/dynamic recrystallization may occur at these conditions. Distinct oscillation could be observed at the strain rate of 10 s^−1^, and a similar phenomenon was observed in relevant work [23,28]. The main reason may be the occurrence of flow localization between the α/β phase at relatively high strain rates. It is worth noting that the flow curve at the strain rate of 10 s^−1^ in Figure 2d shows a rise with the strain increases, which means the work hardening process may still exist at a relatively high temperature and strain rate.

Notably, the peak values of flow stress of the PM-TiAlFeMo alloy in the present work are 20–50 MPa lower than similar titanium alloys [29,30,31] (e.g., Ti60, IMI834, Ti-1100) at the same deformation conditions. Moreover, the difference between these alloys shows more distinct at high strain rates. It may be interpreted as the addition of an Mo element, which could bring in a modified balance of α and β phase in titanium alloys [32], also brings in the favorable workability of PM-TiAlFeMo alloy.

#### 3.1.2. Flow Softening Behavior

**Figure 3:**
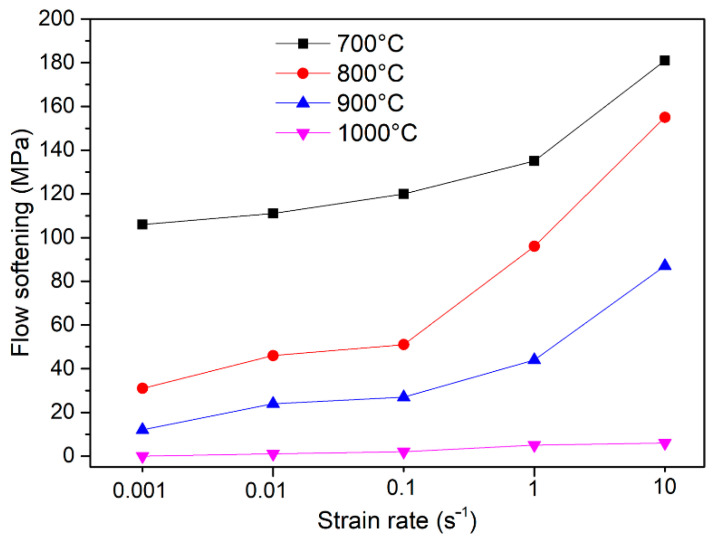
Flow softening degree (∆σ = σ_m_ − σ_0.65_) of the PM-TiAlFeMo alloy deformed at different temperatures and strain rates.

According to Figure 2, it is worth noting that the flow softening degree varies with strain rate and testing temperature. The flow softening degree [21] could be described by Equation (1):

∆σ = σ_m_ − σ_s_ (1)

where the σ_m_ is the peak value of the flow stress, σ_s_ is a relatively steady-state value of stress in the later stage of deformation. According to Figure 2, the relatively steady-state value could be observed near the true strain of 0.65. Hence, the flow softening degree could be obtained by calculating the value of (σ_m_ − σ_0.65_). Figure 3 shows the flow softening degree calculated from the flow curves at various deformation conditions. The flow softening at low temperatures (700, 800 °C) shows more distinct than high temperature (900 °C). Furthermore, the flow softening degree increases with the increase in the strain rate. In general, the dynamic recrystallization and flow localization are the dominant reasons for the flow softening phenomenon during hot deformation in dual-phase titanium alloys [33]. Relevant studies [24,34,35] also suggested that flow softening is mainly due to the rotation of the α phase to the direction perpendicular to the compression axis (CA) in lamellar titanium alloys. Platelet bending/kinking and dynamic globularization are also correlative reasons for flow softening [23,34]. Those factors could be determined in the microstructure observation section. 

### 3.2. Arrhenius-Type Constitutive Modeling

Based on the analysis above, it can be found that the flow behavior of the PM-TiAlFeMo alloy is significantly affected by the deformation temperature and strain rate during hot compression. Constitutive modeling is an important method to model the hot deformation behavior of metallic materials, which could help in predicting and selecting appropriate processing parameters by combining the utilization of processing maps [3]. The Arrhenius-type equation like Equations (2)–(4) are widely used to describe the relationship between flow behavior and deformation parameters [36]:

(2) $$\dot{\varepsilon \;}{=\;A\;\sigma }^{n_{1}}\;\exp[-Q/RT]\;\left(\mathrm{at}\;\mathrm{low}-\mathrm{stress}\;\mathrm{conditions}\right)$$

(3) $$\dot{\varepsilon \;}=\;A\;\exp\;(\beta \sigma )\;\exp[-Q/RT]\;\left(\mathrm{at}\;\mathrm{high}-\mathrm{stress}\;\mathrm{conditions}\right)$$

(4) $$\dot{\varepsilon \;}{=\;A[\sin h(\alpha \sigma )}^{n}]\exp[-Q/RT]\;(\mathrm{at}\;\mathrm{all}\;\mathrm{conditions})\;$$

where the *A*, *n*_1_, *n*, α, and β are constants of materials. Q is the thermal activation energy (kJ/mol) of a certain alloy, *T* is the testing temperature (absolute temperature, K), and R is a universal constant of molar gas. In addition, the value of *α* in Equation (4) is a variable constant, which varies from the deformation parameters. The relevant literature [37,38] proposed that the value of α satisfies the equation: α = β/*n*_1_, which has been applied widely in the Ti–6Al–4V, IMI834, and other titanium alloys.

Taking the natural logarithm of both sides of Equations (2)–(4), respectively, yields:

(5) $$\ln\dot{\varepsilon \;}\;=\;\ln A_{1}\;+\;n_{1}\ln\sigma -Q/RT$$

(6) $$\ln\dot{\varepsilon \;}\;=\;\ln A_{2}\;+\;\beta \sigma \;-\;Q/RT$$

(7) $$\ln[\sin h(\alpha \sigma )]\;=\;\frac{\ln\dot{\varepsilon \;}}{n}\;+\;\frac{Q}{nRT}\;-\;\frac{\ln\;A}{n}$$

**Figure 4:**
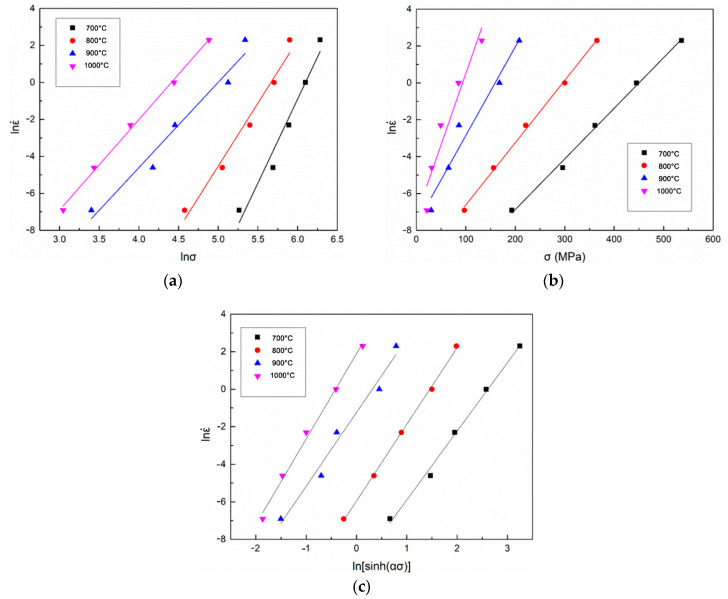
Relationship between the peak stress and strain rate of the PM-TiAlFeMo alloy: (**a**) ln$\dot{\varepsilon }$–lnσ; (**b**) ln$\dot{\varepsilon }$–σ; and (**c**) ln$\dot{\varepsilon }$–ln(sinh(ασ)).

It is clear that a linear correlation could be built between (ln$\dot{\varepsilon }$–lnσ), (ln$\dot{\varepsilon }$ – σ) and (ln$\dot{\varepsilon }$–ln[sinh(ασ)]). The value of *n*_1_, β and *n* could be represented, respectively, by the slope values of these curves at constant temperatures. Figure 4 demonstrates the quantitative correlation of (ln$\dot{\varepsilon }$–lnσ), (ln$\dot{\varepsilon }$–σ) and (ln$\dot{\varepsilon }$–ln[sinh(ασ)]) of the PM-TiAlFeMo alloy. A slight deviation can be observed at relatively high temperature (1000 °C), it may be associated with the phase transformation under this condition. The phase transformation temperature of this alloy is determined as 915 °C. When the testing temperature reached this point, the α phase would transfer to the β phase with the temperature rises. The deformation mechanisms of β (BCC) phase and α (HCP) phase are different, so the phase transformation at relatively high temperature may affect the fit of a constitutive relationship. Based on the slope values of the curves in Figure 4, the value of *n*_1_ and β could be obtained. Therefore, the corresponding value of α under different conditions could be calculated (α = β/*n*_1_), and the average value of α was calculated as 0.0068.

In order to compare the accuracies of those three equations, the correlation coefficient (R or R^2^) [24,30,39] was employed to verify the correlation between (ln$\dot{\varepsilon }$–lnσ), (ln$\dot{\varepsilon }$–σ) and (ln$\dot{\varepsilon }$–ln[sinh(ασ)]). The average values of R^2^ of (ln$\dot{\varepsilon }$–lnσ), (ln$\dot{\varepsilon }$–σ) and (ln$\dot{\varepsilon }$–ln[sinh(ασ)]) are 0.9213, 0.9327 and 0.9681, respectively. This means that the hyperbolic sinh equation (Equation (7)) has better accuracy than the power series equations (Equations (5) and (6)). In addition, Chen et al. [29] introduced the parameter of RSD (relative standard deviation) to quantitatively depict the dispersion of a materials constant in order to reduce the adverse effect of the deformation temperature on the material constants. According to the equation provided in their literature, the RSD values of (ln$\dot{\varepsilon }$–lnσ), (ln$\dot{\varepsilon }$–σ) and (ln$\dot{\varepsilon }$–ln[sinh(ασ)]) were calculated: 12.7%, 11.9% and 3.2%, which are consistent with the results of the coefficient of R^2^.

**Figure 5:**
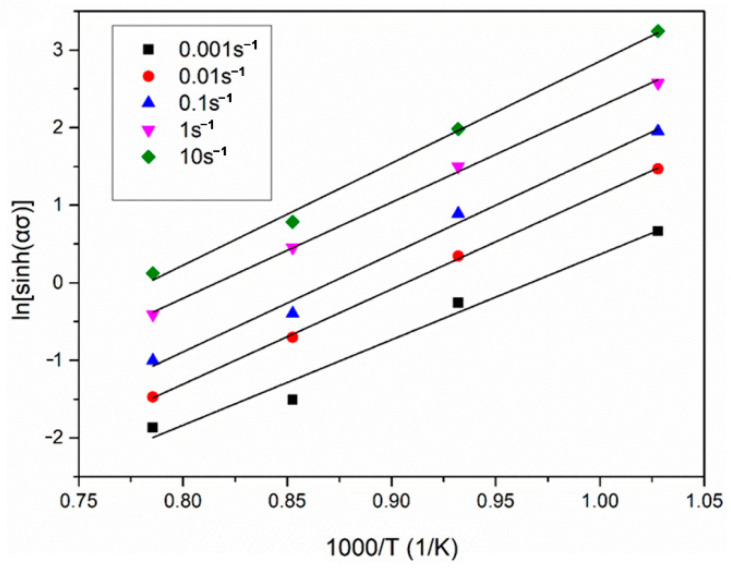
Linear regression analysis of ln[sinh(ασ)]–1/T.

Based on the analysis of accuracy above, Equation (7) was employed to calculate the value of Q of the PM-TiAlFeMo alloy. Taking the partial differentiation of Equation (7) yields:

(8) $$Q\;=\;R{\left\{\frac{\partial \ln\dot{\varepsilon \;}}{\partial \ln[\sin\;h(\alpha \sigma )]}\right\}}_{T}\times \;{\left\{\frac{\partial \ln[\sin h(\alpha \sigma )]}{\partial \left(1/T\right)}\right\}}_{\dot{\varepsilon \;}}$$

where the $\partial \ln\dot{\varepsilon \;}/\partial \ln[\sin h(\alpha \sigma )]$ (as the value of *n*) is the slope value of ln$\dot{\varepsilon }$–ln[sinh(ασ)] linear regression line at a given deformation temperature (Figure 4c). The ∂ln[sinh(ασ)]/∂(1/T) is the slope value of the ln[sinh(ασ)]–1/T linear regression line (Figure 5).

The thermal activation energy (Q) of the alloy under various hot deformation parameters could be calculated based on plotting the diagram of the relationship above. Meanwhile, the value of the constant A could be calculated from the intercept of the linear regression line of ln$\dot{\varepsilon }$–ln[sinh(ασ)]. By means of multiple iterations, value of A, n and Q are calculated: exp (39.84), 6.85 and 413.25 kJ/mol.

In general, the workability of a certain metals relates to the thermal activation energy (Q), where a lower value of Q is favorable for the hot processing of an alloy. Table 2 shows the Q values of several similar titanium alloys. Compared with other similar alloys, the thermal activation energy of the PM-TiAlFeMo alloy in the present work is 100–200 kJ/mol lower than others, which shows better workability. Moreover, the thermal activation energy values of nearly single phased titanium alloys are higher than the dual-phased alloys. A similar result was obtained in Dehghan’s research [40]. Relevant studies [41,42,43] try to explain the difference in activation energy between titanium alloys, which suggest it may be related to the phase composition of the alloy. The high activation energy of a single phased titanium alloy may be due to the high flow stress of the hard phase (α-Ti) [43]. On the contrary, the α + β phase always corresponds to relatively low thermal activation energy [44]. Furthermore, the appropriate porosity and homogeneous structure in PM materials are beneficial to decrease the deformation resistance and activation energy due to the elimination of pores at the initial stage of hot compression. Peng et al. [45] proposed that the addition of elemental Mo could help improve the workability of titanium alloys, which could also be considered the reason for the relatively low thermal activation energy of the PM-TiAlFeMo alloy.

Based on the calculations above, the constitutive equation of the PM-TiAlFeMo alloy could be obtained as follows:

(9) $$\dot{\varepsilon \;}\;=\;\exp\left(39.84\right){\left[\sin h(0.0068\sigma )\right]}^{6.85}\exp(-413.25/RT)$$

Furthermore, the Zener–Hollomon (Z) parameter [47] is another important factor which is employed to describe the effects of the deformation parameters on the flow behavior of metallic materials, as presented in Equation (10):

(10) $$Z\;=\;\dot{\varepsilon \;}\exp(\frac{Q}{RT}{)\;=\;A[\sin h(\alpha \sigma )]}^{n}$$

Taking the logarithm of both sides of Equation (10), which yields:

(11) lnZ = lnA + nln[sinh(ασ)]

**Figure 6:**
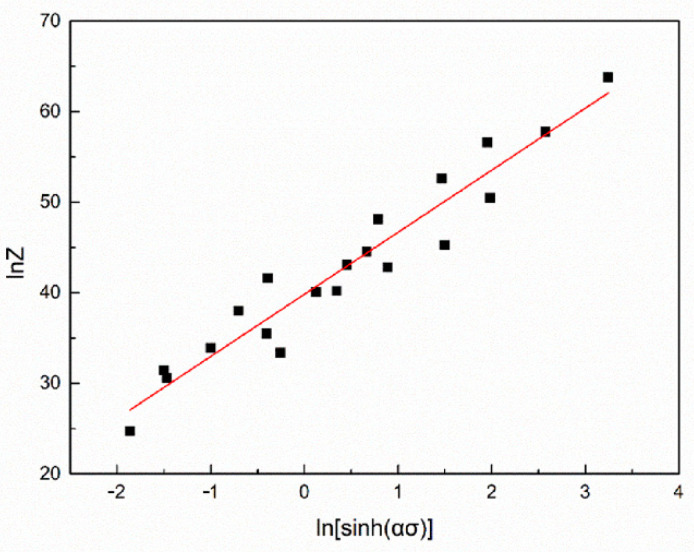
Linear regression analysis of lnZ–ln[sinh(ασ)].

The linear regression curve between lnZ and ln[sinh(ασ)] is illustrated in Figure 6, the calculated value of the R^2^ between lnZ and ln[sinh(ασ)] is 0.9765, which means that the linear fitting correlation between the two factors is good and the hyperbolic sinh equation is reliable to be employed.

**Figure 7:**
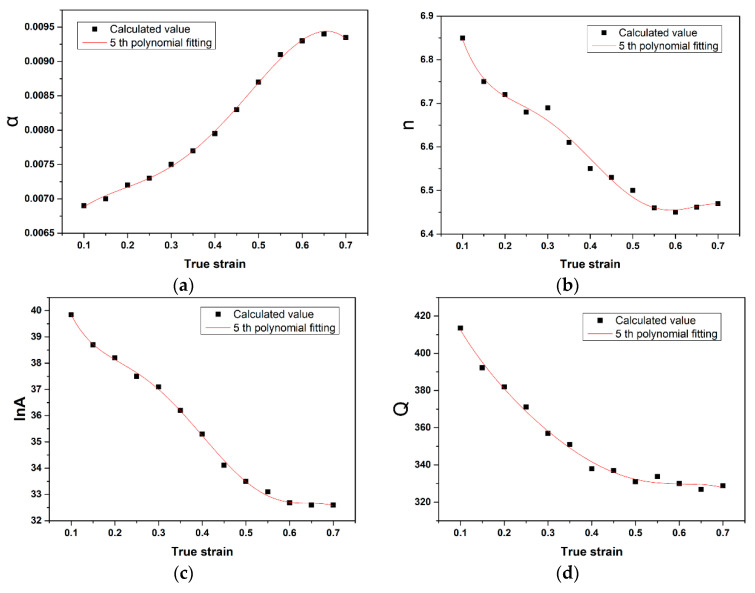
Relationship between the material constants and true strain of the PM-TiAlFeMo alloy by polynomial fitting: (**a**) α; (**b**) *n*; (**c**) ln*A*; and (**d**) Q.

Generally, the effect of strain on the hot deformation behavior of a certain alloy is assumed as insignificant and the factor of strain has not been taken into consideration in Equations (2)–(4). Conversely, the material constants (i.e., ln*A*, *n*, α and Q) are significantly affected by the condition of strain. The prediction of the flow stress and workability of a material is directly affected by the accuracy of the calculation of material constants. Hence, it is significant to improve the accuracy of the calculating process by considering the influence of strain. In the present work, those material constants under different strains were calculated by multiple iterations from 0.1–0.7 with an interval of 0.05, then plot these points in Figure 7. Previous studies [38,48] suggested that the correlations of *ε* and α, *n*, ln*A*, Q could be described by multi-order polynomial functions. Ma et al. [49] built a 4th order polynomial function to describe the material constant of a Ti–15Mo–3Al–2.7Nb–0.2Si alloy, and Ghavam et al. [38] created a 7th order polynomial function to describe the material constants of the IMI834 alloy. However, Cai et al. [50] proposed that the polynomial function with too high an order would overfit and lose the ability of true representation. In order to set an appropriate order for the polynomial functions, the method of trial and error was employed by trying the order from 1 to 8. Consequently, polynomial functions with an order of 5 like Equation (12) were built, which shows good correlation (R^2^ = 0.9761) and low dispersion with the calculated scattered data points in Figure 7. The fitting results for the coefficients of the polynomial functions are shown in Table 3:

(12) $$\{\begin{array}{c} \alpha \;=\;E_{0}+E_{1}\varepsilon \;+\;E_{2}\varepsilon^{2}\;+\;E_{3}\varepsilon^{3}+\;E_{4}\varepsilon^{4}\;+E_{5}\varepsilon^{5}\\ \;n\;=\;F_{0}+F_{1}\varepsilon \;+F_{2}\varepsilon^{2}+F_{3}\varepsilon^{3}+F_{4}\varepsilon^{4}+F_{5}\varepsilon^{5}\\ \ln\;A\;=\;M_{0}+M_{1}+M_{2}\varepsilon^{2}+M_{3}\varepsilon^{3}+M_{4}\varepsilon^{4}+M_{5}\varepsilon^{5}\\ Q\;=\;N_{0}+N_{1}\varepsilon +N_{2}\varepsilon^{2}+N_{3}\varepsilon^{3}+N_{4}\varepsilon^{4}+N_{5}\varepsilon^{5} \end{array}$$

Once the coefficients are revealed by the 5th polynomial fitting, the material constants could be calculated by Equation (12). Then, the predicted value of flow stress (σ_p_) could be obtained by Equation (13). The value of Z could be calculated by Equation (10):

(13) $$\sigma_{p}\;=\;\frac{1}{\alpha }\ln\left\{{\left(\frac{Z}{A}\right)}^{\frac{1}{n}}+{\left[{\left(\frac{Z}{A}\right)}^{\frac{2}{n}}+\;1\right]}^{\frac{1}{2}}\right\}$$

**Figure 8:**
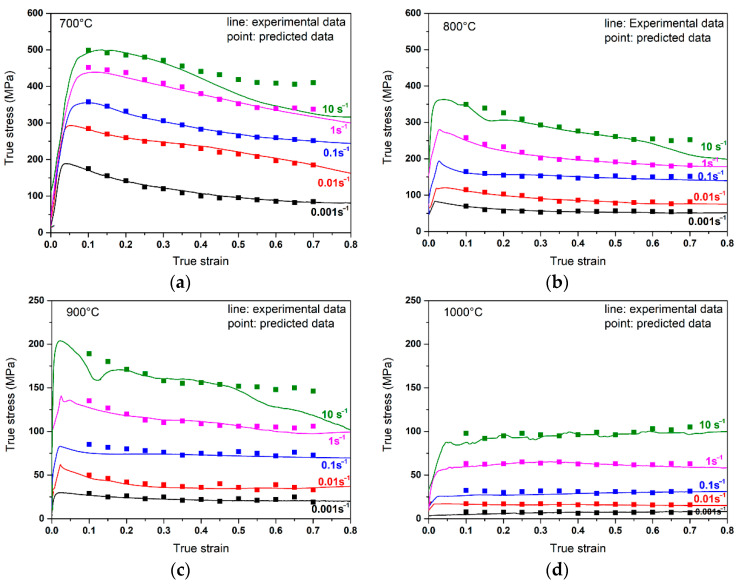
Comparison between the predicted data and experimental data of flow stress at different temperatures and strain rates: (**a**) 700 °C; (**b**) 800 °C; (**c**) 900 °C; and (**d**) 1000 °C.

As shown in Figure 8, the predicted values of flow stress under various deformation conditions were calculated and compared with the experimental data. The predicted values coincide well with the experimental data in most deformation parameters. Only a slight deviation can be observed at 700 and 900 °C/10 s^−1^. This may come from the unstable flow, micro-cracks, and flow localization at the relatively high strain rates.

Relevant literature [24,51] suggested that the predictability of the constitutive equation could be verified by the parameter of average absolute error (AARE%). In general, the AARE% is considered as an unbiased statistical parameter. The value of AARE% could be calculated by Equation (14):

(14) $$\mathrm{AARE}\%\;=\;\frac{1}{M}\sum_{i=1}^{M}\left|\frac{\sigma_{e}{\;-\;\sigma }_{p}}{\sigma_{e}}\right|\times 100$$

where σ_e_, σ_p_ is the experimental and predicted value of flow stress, and M is the number of data points, which is 260 in the present work. The AARE% of the PM-TiAlFeMo alloy is calculated: 5.21%. Ideally, the value of AARE% is “0”. In practice, a smaller AARE% represents a better correlation between experimental and predicted data. A comparison between the present work and relevant literature is shown in Table 4, it can be confirmed that the Arrhenius-type model shows better predictability to estimate the flow stress of the PM-TiAlFeMo alloy compared with similar titanium alloys. Wang et al. [51] introduced a modified parameter (ζ) into the original constitutive model to reduce the error of prediction. By introducing the modified parameter, the AARE% of the Ti-10-2-3 alloy in their research decreased from 5.61% to 2.81%. However, the AARE% of the PM-TiAlFeMo alloy in the present work only changed from 5.21 to 5.198% using the parameter (ζ), which indicates the modified parameter shows a better accuracy for the specific alloy in Wang’s study rather than the PM-TiAlFeMo alloy in our work.

### 3.3. Processing Map

A processing map is an important tool that is always employed to predict the hot forming process and flow behavior of metallic materials by analyzing the deformation instability information during hot deformation. Generally, a processing map could be drawn by combining the flow instability information and power dissipation diagram. Equation (15) is widely employed to calculate the value of power dissipation efficiency (*η*) based on the dynamic materials model (DMM) established by Prasad et al. [53]:

(15) $$\eta \;=\;\frac{2m}{m\;+\;1}$$

The *m* is the strain rate sensitivity, which represents the energy input during hot deformation is consumed by microstructure evolution rather than heat dissipation. The value of m could be defined by Equation (16):

(16) $$m\;=\;\frac{\partial ln\sigma }{\partial ln\dot{\varepsilon \;}}$$

**Figure 9:**
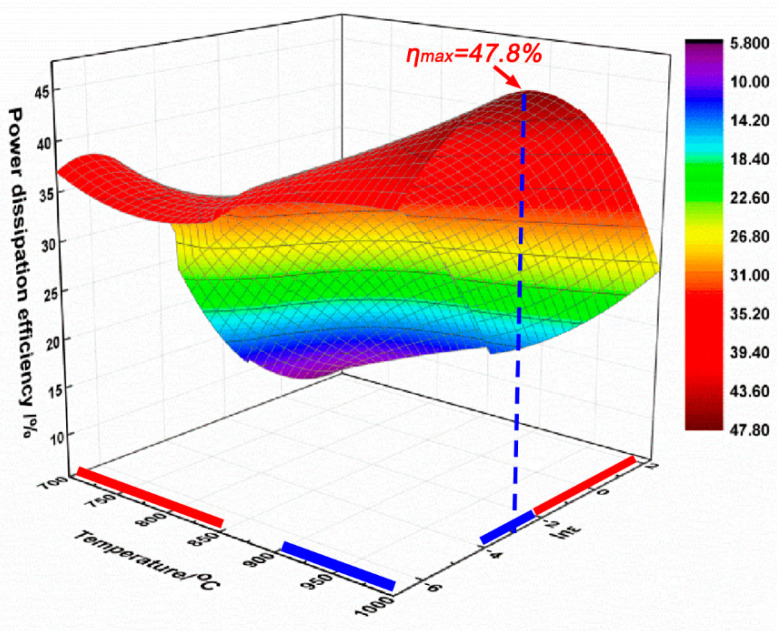
3D power dissipation efficiency map of the PM-TiAlFeMo alloy.

Figure 9 shows the 3D power dissipation efficiency map of the PM-TiAlFeMo alloy. It is clear that the value of *η* changes insignificantly with temperature but significantly with the strain rate. The *η* ranges from 5.8 to 47.8%, the minimum value and maximum value appears at the condition of 700 and 1000 °C/0.01 s^−1^, respectively. In general, the relatively higher value of *η* represents the better workability of an alloy. As shown in Figure 9, the ranges marked by blue bars along the *x* and *y* axes are relatively secure processing regions, and in opposition, the red bars are insecure.

On the other hand, a few unpredictable defects (e.g., micro-cracks, flow localization) may cause unstable flow during the hot process, which significantly affects the secure region in the processing map. Prasad et al. [53] proposed the criterion for the flow instability, as shown in Equation (17):

(17) $$\xi \left(\dot{\varepsilon \;}\right)\;=\;\frac{\partial \ln[m/(m\;+\;1)]}{\partial ln\dot{\varepsilon \;}}\;+\;m$$

when the value of *ξ* < 0, it is considered as the unstable flow.

**Figure 10:**
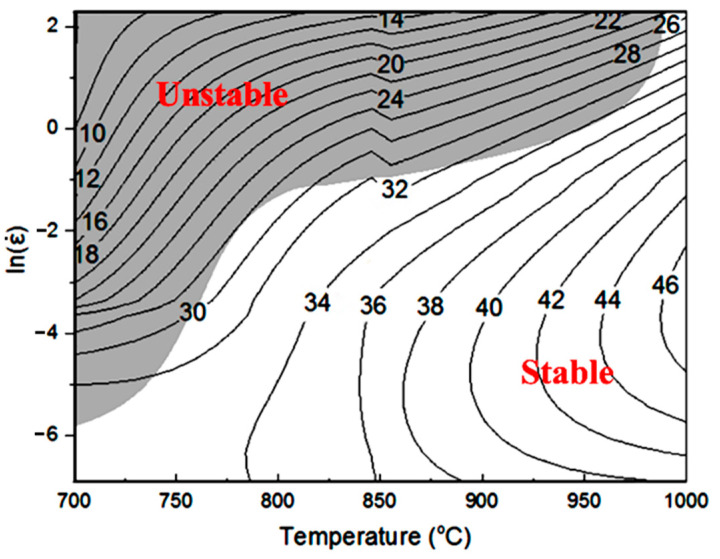
Processing map of the PM-TiAlFeMo alloy.

By superimposing the unstable zone determined by Equation (17) on the power dissipation diagram, the processing map could be drawn considering the strain rate, deformation temperature and power dissipation efficiency (as shown in Figure 10). The dark region with a relatively low *η* is identified as flow instability zone, and the white region means flow stability zone. The range of 900–1000 °C/0.001 s^−1^–0.1 s^−1^ is a relatively secure zone that could be chosen in practical processing. Sen et al. [54] suggest that the high-temperature deformation mechanism under various deformation parameters is closely related to the power dissipation efficiency. The peak value of *η* often corresponds to the favorable deformation mechanisms, where dynamic recovery, dynamic recrystallization and super-plasticity are dominant mechanisms for these areas [27]. Conversely, unstable flow (e.g., kinking, breaking, and flow localization) may occur at a low strain rate/low temperature (0.01–10 s^−1^/700–850 °C) with a relatively low *η* value, which needs to be avoided during hot processing.

### 3.4. Deformation Microstructures

It is known that the flow stability/instability and high/low power dissipation coefficient in the processing map represent various microstructure evolutions during hot processing. Hence, it is significant to verify those analyses about above deformation mechanisms by microstructure observation. Moreover, it is also an effective method to verify the accuracy of the DMM model and choose the optimal processing parameters in the practical hot working process.

#### 3.4.1. Effects of Temperature on Deformation Microstructure

**Figure 11:**
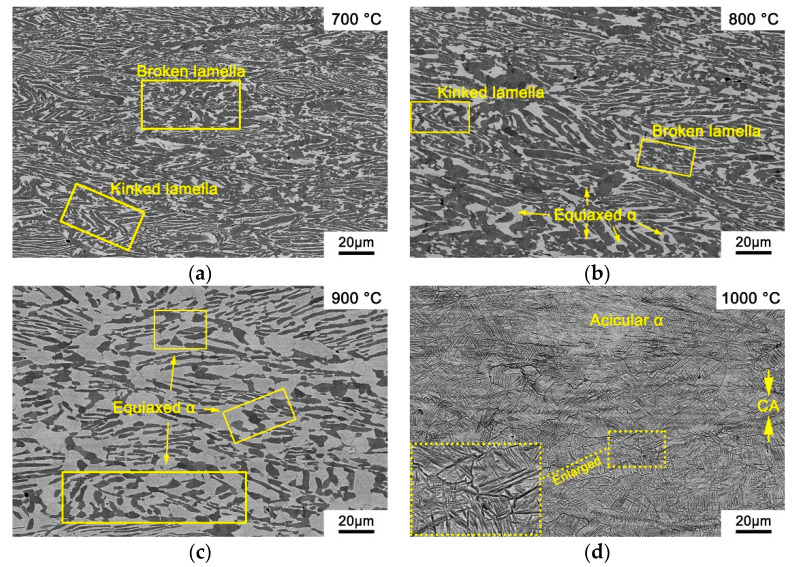
Microstructures of the PM-TiAlFeMo alloy deformed at the strain rate of 0.001 s^−1^: (**a**) 700 °C; (**b**) 800 °C; (**c**) 900 °C; and (**d**) 1000 °C.

The deformation microstructure of the PM-TiAlFeMo alloy deformed at the condition of 0.001 s^−1^/700 °C–1000 °C is illustrated in Figure 11. It is worth noting that the pores in the as-sintered PM-TiAlFeMo alloy (Figure 1) have been eliminated, which agrees with the discussion of correlation between porosity and activation energy (Section 3.2). As shown in Figure 11a,b, the originally lamellar microstructure which is parallel/perpendicular or angled (e.g., 45°) to the compression axis (CA) has been kinked/broken or rotated after compression at relatively low deformation temperatures. For HCP Ti, the dislocation climbing and cross-slipping will be restricted at relatively low temperatures. Therefore, the energy stored during compression could not be released at relatively low strain rates and temperatures. Consequently, the breaking, rotation and kinking of microstructure will occur as the strain increases and accumulates. As shown in Figure 11b, the equiaxed α phase could be observed in partial areas, which means dynamic globularization occurred at 800 °C. The globularization in Figure 11c (900 °C) shows to be more distinct compared with Figure 11b. A similar result was observed in the Ti600 alloy [23], which is well consistent with the current result that higher deformation temperature help globularization to occur during hot deformation. When the deformation temperature exceeds T_β_, the original lamellar α + β microstructure transforms into nearly single β during hot deformation (Figure 11d). The deformation driving force rapidly increases at high temperature, the agglomeration and growth of β grains may occur more easily under this condition [23,55]. Ultrafine acicular α phase precipitated in the β phase during water quenching after hot compression, so the microstructure after deformed at 1000 °C showed a mixture of the acicular α phase and retained β. Moreover, small-size recrystallization grains could be observed in the junction of coarse deformed β grain boundaries (as marked by frame and presented in the enlarged figure at the lower-left corner in Figure 11d), which could be interpreted that recrystallization prefers to occur in those areas [21]. When hot working is performed at the temperature above T_β_, dislocation migration will be enabled by the high self-diffusivity of BCC Ti with the increase in temperature, and the stored energy will decrease, which help dynamic recovery, dynamic recrystallization and grain growth to occur. It is consistent with the discussion above (Section 3.3), where the peak value of power dissipation efficiency at 1000 °C/0.01 s^−1^ often corresponds to the dynamic recovery and recrystallization.

**Figure 12:**
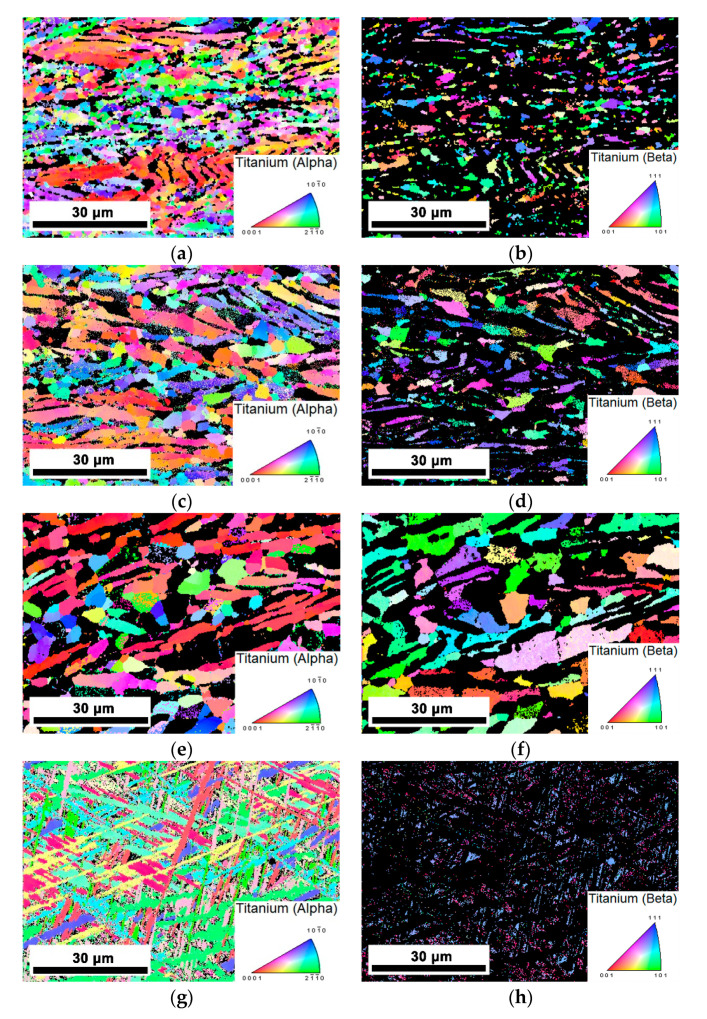
IPF maps of the PM-TiAlFeMo alloy deformed at the strain rate of 0.001 s^−1^: (**a**,**b**) 700 °C; (**c**,**d**) 800 °C; (**e**,**f**) 900 °C; and (**g**,**h**) 1000 °C.

The inverse pole figures of the PM-TiAlMoFe alloy deformed at the condition of 0.001 s^−1^/700 °C–1000 °C are shown in Figure 12. Breaking and globularization could be observed at the temperature of (α + β) region (Figure 12a,c,e), the grain size obviously increases as deformation temperature increases. The deformation microstructure exhibits distinct preferred orientation (α-0001)/(β-101)/(β-111), which indicates that the preferential deformation will occur along the slip system during hot compression. For titanium alloys, β phase (BCC) has twelve {101} <111> slip systems which are much more than three {0001} <11–20> slip systems in α phase (HCP) in titanium alloy [56], confirming that the workability of the PM-TiAlFeMo alloy is better at a relatively high temperature with a high volume fraction of the β phase.

#### 3.4.2. Effects of Strain Rate on Deformation Microstructure

**Figure 13:**
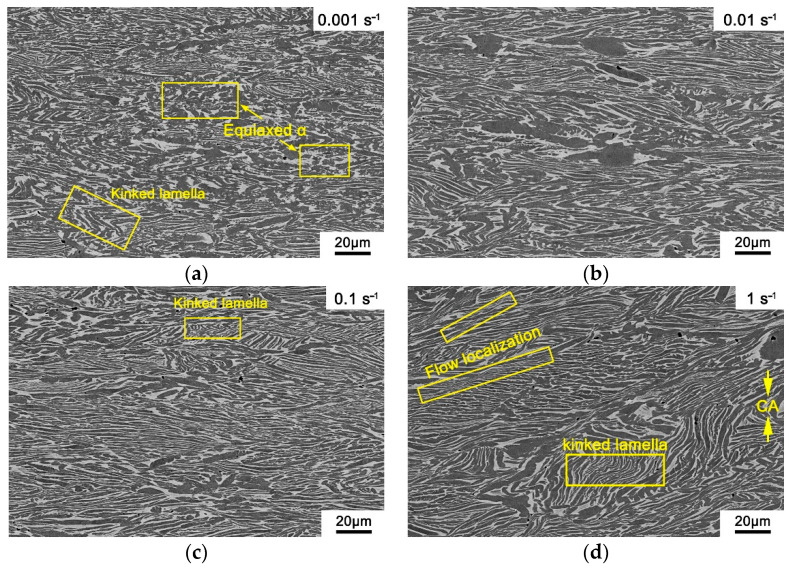
Microstructures of the PM-TiAlFeMo alloy deformed at the temperature of 700 °C: (**a**) 0.001 s^−1^; (**b**) 0.01 s^−1^; (**c**) 0.1 s^−1^; and (**d**) 1 s^−1^.

The deformation microstructure of the PM-TiAlFeMo alloy deformed at the condition of 700 °C/0.001 s^−1^–1 s^−1^ (temperature/strain rate) is illustrated in Figure 13. Kinked lamellar α/β phase and equiaxed α could be observed (marked by frame in Figure 13a), which means the microstructure has enough time to spheroidize at a relatively low strain rate (0.001 s^−1^). The primary α phase is distinctly elongated in Figure 13b, and the elongated α phase is perpendicular to the compressive axis (CA). Furthermore, the elongated α phase becomes thinner, broken, or kinked during deformation when the strain rate is 0.1 s^−1^ (Figure 13c). It may be interpreted as the increasing dislocation accumulation rate and dislocation density at a relatively high strain rate [23]. When the strain rate increases to 1 s^−1^, a kinked lamellar phase and flow localization could be observed in the deformation microstructure (Figure 13d). This coincides with the analysis of a deformation mechanism in Section 3.1.1, where the flow localization and kinking may occur at a relatively high strain rate. Similar results were observed in the relevant literature [41], indicating a partial high heat during deformation and low thermal conductivity of the alloy could result in inhomogeneous deformation. Furthermore, the high strain rate is unfavorable to the evolution from the substructure to stable recrystallized grains formed by dislocation accumulation, which also leads to inhomogeneous deformations (e.g., kinking or flow localization). It is consistent with the high *η* at a relatively low temperature/high strain rate in the processing map, which often corresponds to the unfavorable area for hot deformation.

## 4. Conclusions

The thermal activation energy (Q) of the PM-TiAlFeMo alloy is 413.25 kJ/mol. The relationship between the strain rate, temperature and peak value of the flow stress could be described by a hyperbolic sinh Arrhenius equation, which has good accuracy in describing the hot deformation behavior of the PM-TiAlFeMo alloy:

(18) $$\dot{\varepsilon \;}=\;\exp(39.84)[\sin h(0{.0068\sigma )]}^{6.85}\exp(-413.25/RT)$$

The PM-TiAlFeMo alloy is suitable to be hot processed at the condition of (900–1000 °C/0.001–0.1 s^−1^), which is attributed to the dynamic recovery and dynamic recrystallization occurring in this range. However, the flow localization caused by inhomogeneous deformation makes the area of (700–850 °C/0.01–10 s^−1^) unsuitable for performing hot deformation.

The flow behavior of the PM-TiAlFeMo alloy is significantly affected by the deformation temperature and strain rate. The mechanisms of flow softening at relatively low temperature (below T_β_) are mainly kinking, breaking and dynamic globularization. Dynamic recovery and recrystallization are dominant mechanisms at relatively high temperatures. Kinking and flow localization occur at relatively high strain rates, while dynamic globularizations have sufficient time to nucleate and grow at relatively low strain rates.

## Figures and Tables

**Table 1:** Detailed information of raw elemental powders.

Powders	Purity (wt.%)	O (wt.%)	Other Impurity (wt.%)	Particle Size, D50 (μm)	Producing Methods	Producer
Ti	99.744 ± 0.015	0.22 ± 0.01	0.036 ± 0.005	67.5 ± 0.2	Hydrogenation-dehydrogenation	TiTd Metal Materials Co., Ltd., Changsha, China
Al	99.718 ± 0.015	0.27 ± 0.01	0.012 ± 0.005	21.3 ± 0.2	Gas atomization	
Fe	99.811 ± 0.015	0.18 ± 0.01	0.009 ± 0.005	25.3 ± 0.2	Deoxidization	
Mo	99.729 ± 0.015	0.26 ± 0.01	0.011 ± 0.005	6.18 ± 0.2	Deoxidization	

**Table 2:** Comparison of the value of Q among several similar titanium alloys.

Alloy	Refs.	Q (kJ/mol)	Type	Deformation Temperature (°C)	Initial Microstructure
Ti60	[43]	591	Near α	900–1000	Acicular
IMI834	[38]	557	α + β	800–1000	Transformed β
Ti-6242S	[46]	623	Near α	816–955	Lamellar
TiAlFeMo	Present work	413.25	α + β	700–1000	Lamellar

**Table 3:** The fitting results for the coefficients of the polynomial functions.

Subscript of Coefficient	Material Constants			
	α (E_x_)	*n* (F_x_)	ln*A* (*M*_x_)	Q (*N*_x_)
0	0.00616	7.40278	46.65846	464.1805
1	0.0114	−9.67458	−120.575	−687.345
2	−0.05417	54.39862	702.7041	2179.603
3	0.13092	−150.532	−2050.21	−5502.86
4	−0.09441	190.761	2683.395	7744.687
5	−0.00302	−89.1704	−1279.64	−4137.25

**Table 4:** Comparison of AARE% between the PM-TiAlFeMo alloy and similar alloys.

Alloy	Refs.	Type	Deformation Temperature (°C)	Strain Rate (s^−1^)	AARE%
IMI834	[30]	near α	850–1060	0.0003–1	10.43
Ti-6242S	[24]	near α	850–1000	0.001–1	9.98
Ti60	[39]	near α	970–1120	0.01–10	8.45
Ti-6Al-4V	[50]	α + β	800–1050	0.0005–1	9.06
Ti–6Al–7Nb	[52]	α + β	850–1000	0.0025–0.25	5.53
Ti–5Al–2Fe–3Mo	Present work	α + β	700–1000	0.001–10	5.21

## Data Availability

The data presented in this study are available from the corresponding author on reasonable request. The data are not publicly available due to privacy.
